# Kea, bird of versatility. Kea parrots (*Nestor notabilis*) show high behavioural flexibility in solving a demonstrated sequence task

**DOI:** 10.1007/s10336-023-02127-y

**Published:** 2023-12-16

**Authors:** Elisabeth Suwandschieff, Ludwig Huber, Thomas Bugnyar, Raoul Schwing

**Affiliations:** 1 Comparative Cognition, Messerli Research Institute, University of Veterinary Medicine Vienna, Vienna, Austria; 2 Department of Cognitive Biology, University of Vienna, Vienna, Austria

**Keywords:** Kea (*Nestor notabilis*), Behavioural flexibility, Sequence-task, Problem-solving

## Abstract

Social learning is an important aspect of dealing with the complexity of life. The transmission of information via the observation of other individuals is a cost-effective way of acquiring information. It is widespread within the animal kingdom but may differ strongly in the social learning mechanisms applied by the divergent species. Here we tested eighteen Kea (*Nestor notabilis*) parrots on their propensity to socially learn, and imitate, a demonstrated sequence of steps necessary to open an apparatus containing food. The demonstration by a conspecific led to more successful openings by observer birds, than control birds without a demonstration. However, all successful individuals showed great variation in their response topography and abandoned faithfully copying the task in favour of exploration. While the results provide little evidence for motor imitation they do provide further evidence for kea’s propensity towards exploration and rapidly shifting solving strategies, indicative of behavioural flexibility.

**Supplementary Information:**

The online version contains supplementary material available at 10.1007/s10336-023-02127-y.

## Abstract (de)

**Keas, Vögel der Vielseitigkeit. Kea-Papageien (*Nestor notabilis*) zeigen eine hohe Verhaltensflexibilität bei der Lösung einer demonstrierten Sequenzaufgabe.**

Soziales Lernen ist ein wichtiger Aspekt im Umgang mit der Komplexität des Lebens. Das Erlangen von Informationen durch die Beobachtung Anderer ist eine effiziente Möglichkeit der Informationsbeschaffung. Diese Art des Lernens ist im Tierreich weit verbreitet, kann sich jedoch hinsichtlich der unterschiedlichen sozialen Lernmechanismen, welcher sich die verschiedenen Arten bedienen, stark unterscheiden. Achtzehn Kea-Papageien (*Nestor notabilis*) wurden auf soziales Lernen, im Speziellen auf imitatives Lernen einer demonstrierten Abfolge von Schritten zum Öffnen eines mit Futter gefüllten Apparats, getestet. Die Resultate ergaben, dass eine Demonstration durch einen Artgenossen bei Beobachtervögeln zu erfolgreicheren Lösungsansätzen führten als bei Kontrollvögeln welche keine Demonstration erhielten. Allerdings zeigten alle erfolgreichen Individuen große Unterschiede in der Topographie ihrer Reaktion, der genauen Abfolge an Bewegungen. Ebenfalls gaben sie das getreue Kopieren der Aufgabe zugunsten von Erkundungs- und/oder individuellen Lösungsstrategien auf. Insgesamt zeigten unsere Ergebnisse, dass Keas starke Verhaltensvariabilität und Flexibilität zeigen, wenn sie eine komplexe motorische Aufgabe lösen. Damit stellen sie ein großartiges Modellsystem zur Untersuchung der Verhaltensflexibilität dar.

## Introduction

Social learning can be defined “as a change in behavior that follows the observation of another (typically a conspecific) perform a similar behavior, the products of the behavior, or even the products alone” (Zentall 2012: 114). Which type of information is acquired through observation may differ strongly and is reflected in so-called social learning mechanisms. Motivational factors such as social facilitation (Zajonc 1965) promote the acquisition of information or change in behaviour by the observer via the mere presence of a demonstrator (Zentall 2001). Perceptual factors such as local (Roberts 1941) or stimulus enhancement (Galef 1988) can facilitate information acquisition by drawing the attention of the observer to either the location or stimulus of importance (Zentall 2001). In emulation (Tomasello 1998) the results of a demonstrated behaviour affect the observer who may strive to generate the same effect on the environment/objects as the demonstration did, without necessarily understanding the actions or reproducing the behaviour. Imitation is a specific learning mechanism, defined by a high degree of copying fidelity/response matching (Whiten and Ham 1992; Whiten et al. 2009), and cannot be explained by motivational, perceptual, or attentional factors alone (Zentall 2012), for a comprehensive overview of social learning mechanisms see Hoppitt and Laland (2013). Social learning is taxonomically widespread, ranging from insects to birds and mammals, possibly because it is a cost-effective way of acquiring information. Yet, social learning is not always advantageous (Giraldeau et al. 2002; Garcia-Nisa et al. 2023) and different social learning mechanisms may have different thresholds in this respect.

Great apes, for instance, seem to be less prone to show imitation than other forms of social learning like emulation (Horner and Whiten 2005; Tennie et al. 2006; Clay and Tennie 2018). On the other hand, Marmosets (*Callithrix jacchus*) (Bugnyar and Huber 1997; Voelkl and Huber 2000, 2007) and dogs (*Canis familiaris*) (Huber et al. 2009, 2018, 2020) show high-fidelity imitation despite not being closely related to humans (*Homo imitans* as proposed by Meltzoff 1988). This suggests that high-fidelity imitation may be driven by natural ecology and social structure rather than phylogenetic relatedness. In fact, experimental evidence has illustrated that various avian species, namely budgerigars (*Melopsittacus undulates*) (Dawson and Foss 1965; Heyes and Saggerson 2002), European starlings (*Sturnus vulgaris*) (Fawcett et al. 2002), Japanese Quail (*Coturnix japonica*) (Akins and Zentall 1998; Akins et al. 2002), Common Ravens (*Corvus corax*) (Loretto et al. 2020) and Pigeons (*Columba livia*) (Nguyen et al. 2005) show (simple forms) of motor imitation.

Parrots are renowned for their technical intelligence, vocal mimicry and social learning capacities (Pepperberg and Funk 1990; Huber et al. 2001; Funk 2002; Huber and Gajdon 2006; Werdenich and Huber 2006; Auersperg et al. 2009, 2011, 2012, 2014; Miyata et al. 2011; Goodman et al. 2018; Klump et al. 2021; Smith et al. 2022). Yet surprisingly few parrot species have been tested on their motor imitation skills (budgerigars: Dawson and Foss 1965; Galef et al. 1986; Heyes and Saggerson 2002; grey parrots (*Psittacus erithacus*): Moore 1992; kea (*Nestor notabilis*): Huber et al. 2001; Suwandschieff et al. 2023; Goffin cockatoos (*Cacatua goffiniana*): Auersperg et al. 2012), revealing mixed results. Whereas most studies find evidence for motor imitation, the studies on kea remained inconclusive.

Kea (*Nestor notabilis*) possess well-developed technical skills (Huber and Gajdon 2006), have long lifespans with multiple reproductive cycles, extended juvenile periods accompanied by considerable in-group tolerance, are highly neophilic and exploratory (Diamond and Bond 1999). They also have a very large number of documented food sources (Brejaart 1988; Clarke 1971; O’Donnell and Dilks 1994) many of which need to be extracted, which strongly suggest transfer of knowledge between individuals. All these characteristics facilitate the development of social learning (Gajdon et al. 2004), yet experimental evidence for imitation in this species is still missing. Therefore, we tested kea, for their social learning skills in a demonstrated sequence task. Specifically, we aimed at exploring kea’s imitative social learning capacities. We hypothesised that when confronted with a relatively complex two-step task, kea would pay attention to, and copy the behaviour of, a skilled conspecific. We thus predicted that observers would preferentially use the demonstrated opening side, sequence and colour whereas non-observing control individuals would apply trial-and-error learning to solve the task.

## Method

### Subjects

Eighteen kea from the Haidlhof Research Station (Bad Vöslau) participated in this study. All individuals were group-housed in an outdoor aviary equipped with perches, nesting areas, ponds, and various enrichment. All birds were fed three times a day, had access to water ad libitum and were not food deprived for testing. All individuals had prior experience with experimental testing and participated on a voluntary basis in the task. The testing compartment at the Haidlhof Research Station can be visually separated from the rest of the aviary and can be further divided into two different areas. The individuals were assigned to test groups of three and five individuals and two control groups of five individuals each. The distribution was sex and age balanced, for details see Table 1 of the supplementary material.

### Apparatus

**Fig. 1:**
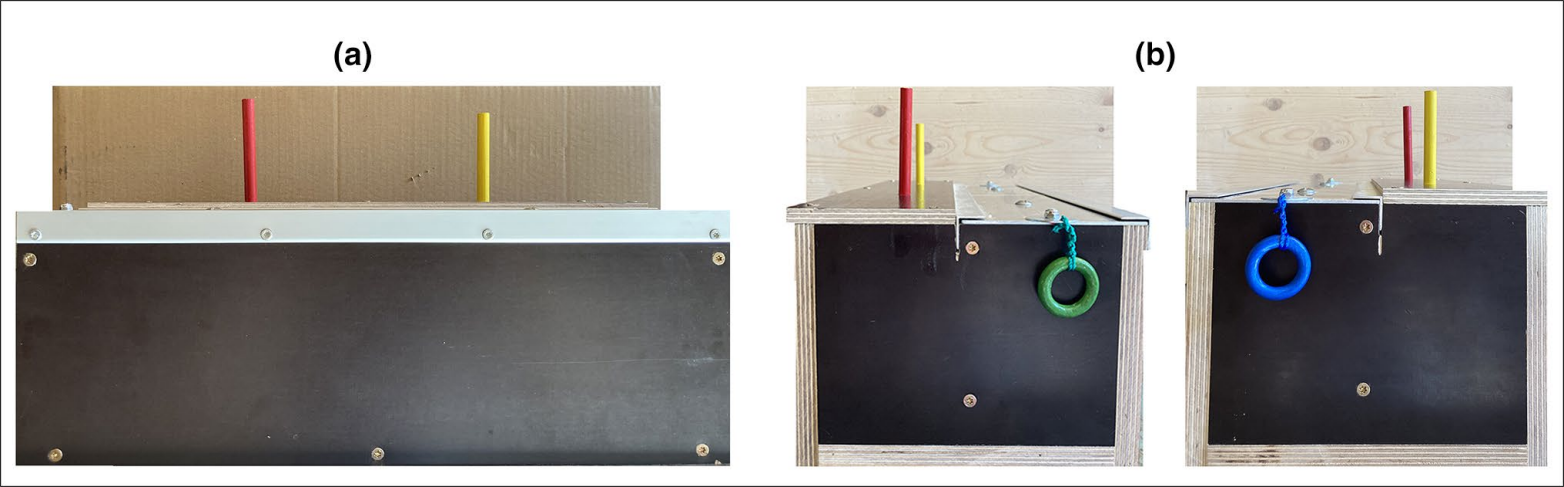
Test box **a** front view of two pins **b** side view of green and blue ring respectively

A rectangular box (44 × 18 × 18 cm) with two aluminium sliding lids, two pins, two strings and two rings served as the test box, see Fig. 1. The test box was designed to provide a sequence function, requiring the subjects to pull a pin on top of the box, to then be able to open the opposite sliding lid by pulling a ring attached at the side of the box. The adjacent sliding lid remained locked. Hence, the only solving sequences for the test box were left pin–right ring or right pin–left ring. Electronics were added inside the box to provide the sequence function. If no pin or both pins were pulled the mechanism locked both lids and no rewards could be retrieved. Manual locking keys were added to provide a full and partial locking function for demonstrator training and demonstration sessions. The box was divided into equally sized reward sections underneath the sliding lids and a GoPro fixture was attached at the base.

The pins were coloured in red and yellow, which is on the preferred spectrum for kea, and the strings and rings in green and blue (less preferred spectrum) respectively (Weser and Ross 2013). Each side and sequence had one preferred and one less preferred colour pairing, minimising the potential for a side bias based on colour preference alone. To increase salience, only those parts that needed direct manipulation were coloured, i.e., the pins, strings, and rings.

### Procedure

To minimize the effects of individual learning over social learning, all test sessions consisted of one trial only. Each session/trial was terminated either after successful reward retrieval or a maximum of two minutes of exploration—i.e. pulling and touching different ring-pin combinations without opening success. A stopwatch was used to track the two minutes and all sessions were terminated by removing the individuals from the test compartment once either criterion (removal response or time-out) was met. Subjects from test groups received two experimental phases, whereas subjects from control groups received three phases (see below). In all phases, both sides of the box were baited and each sequence was rewarded. However, removing a pin and pulling on the wrong ring, pulling both pins or pulling on the rings without having removed the respective pin did not lead to any reward (as the doors were locked). Altogether four sessions (of one trial each) were tested on three consecutive days. On the first day, phase one (consisting of only one session) was tested in the morning and the first session of phase two (consisting of three sessions) in the afternoon. On the second and third day, each individual only received a morning test session of session two and three of phase two respectively.

The experimenter wore mirrored sunglasses (as has been applied before by Bastos and Taylor 2020; Suwandschieff et al. 2023) and remained silent during testing (excluding direct commands such as “enter”, “exit” etc.) to avoid unintentional cueing.

**Phase 1: Forced failure task to all birds** Pins were removed on this occasion and the test box could not be opened. All individuals (of all experimental groups) received access to the test box and were allowed to try and open it for two minutes. As the pins were missing all individuals were forced to fail this task. After two minutes passed the individual exited the testing compartment and the session was completed. This phase was introduced to prime individuals on the non-functioning of the task, focusing the attention towards the essential pins and increasing the motivation to follow a demonstration.

**Phase 2: Non-demonstrated task Control Group (CG)** Control group individuals were allowed to try and solve the task by trial-and-error for three sessions (on three consecutive days).

**Phase 2: Demonstrated task Test Groups (TG)** Test group individuals received three demonstration sessions of three trials each followed by three test sessions of one trial each (test following demonstration session in direct succession) on three consecutive days. Side/sequence and demonstrator assignment were counterbalanced across the two groups (see Table 1 of the supplementary material).

**Phase 3: Demonstrated task Control Test (CGTest)** In the third phase, former control group birds (minus the two demonstrator birds) were randomly assigned to the two demonstrators and were tested again, this time as observer individuals. The test setup was identical to the phase two demonstrated task of the test group.

### Data scoring and analysis

All experiments were videotaped from two sides, behind the observation compartment and directly above the test box (GoPro) within the test compartment, for the exact setup see Fig. 1 of the supplementary material. All GoPro videos of the test sessions were scored/coded with Solomon Coder (version beta 19.08.02) and one independent rater (blind to the study) scored 10% of all videos. Interobserver reliability was tested with Cohen’s Kappa for the categorical (*k* = 0.93) and Intraclass Correlation Coefficient for the numerical data (ICC = 0.711, approach duration; ICC = 0.999, response duration; ICC = 1, solving latency), for more information see the supplementary material.

A total of 74 sessions were analysed. Two individuals did not participate in two sessions and one session respectively, all other individuals completed all three of their test sessions. A binomial test was applied to compare the success rate of the test versus the control group, using the proportion of successful control birds as the baseline chance level of solving the setup spontaneously without demonstration. All other analyses were strictly descriptive.

## Results

After all subjects had experienced a non-functional apparatus (phase 1), they were allowed to engage in the task with or without prior demonstration of a skilled conspecific (phase 2). In sum, four individuals from the test group (out of a total of 8) successfully solved the task and two individuals from the control group (out of a total of 10). The exact binomial test resulted in a nearly significant trend between the test and control group (*x* = 4, *n* = 8, *p* = 2/10; *p-value* = 0.056).

The control group individuals participated in an additional round of testing (phase 3), in which they received a demonstration prior to getting access to the task again. Four individuals who had failed to solve the task without receiving a demonstration successfully solved the task after receiving a demonstration. In contrast, one of the individuals from the control test group, who had solved the task without receiving a demonstration (in the control group round), did not solve the task after receiving a demonstration (in the control test group round). Assessing the birds’ performance after receiving a demonstration (test group plus control test group) *versus* without a demonstration (control group), the exact binomial test revealed a significant difference between the groups with the test groups performing much better on average than the control group (*x* = 8, *n* = 16, *p* = 2/10; *p-value* = 0.007). When comparing the within-subject design of the control group *versus* the control test group (repeated measure) twice as many individuals successfully solved the task after having seen a demonstration (*x* = 4, *n* = 8, *p* = 2/10; *p-value* = 0.056). Half of the successful individuals (4/8) that saw a demonstration solved the task via the opposite (non-demonstrated) sequence and later reversed to the demonstrated sequence in subsequent sessions, see Table 1.

**Table 1:** Individuals that successfully solved the task per experimental group

Name	Sex	Age	Raised	Group	No of successful sessions (out of 3)	Demonstrator	Applied demonstrated sequence	Applied non demonstrated sequence	Stayed consistent for no of sessions	Switched in session no
Frowin	Male	17	Parent	Control	3	NA	NA	NA	3/3	NA
Papu	Female	8	Hand	Control	1	NA	NA	NA	1/1	NA
John*	Male	22	Parent	Test	3	Frowin	in Session 1 and 2	in Session 3	2/3	3
Tai*	Female	3	Parent	Test	2	Frowin	in Session 1 and 3	Never	2/2	NA
Coco	Female	14	Hand	Test	2	Paul	in Session 2	In Session 1	1/2	2
Pick	Male	17	Hand	Test	2	Paul	Never	In Session 1 and 2	2/2	NA
Sunny	Female	14	Hand	Control Test	1	Paul	Never	In Session 1	1/1	NA
Skipper	Male	4	Hand	Control Test	2	Paul	in Session 2 and 3	Never	2/2	NA
Fay	Female	5	Parent	Control Test	1	Frowin	in Session 1	Never	1/1	NA
Plume	Female	14	Hand	Control Test	2	Frowin	in Session 3	In Session 1	1/2	3

Table indicates name, sex, age, rearing, and experimental group. Number of successful sessions out of three test sessions, assigned demonstrator, application of demonstrated sequence and according session information, application of non-demonstrated sequence and according session information, count of consistent opening sequence out of three test sessions, and number of switches in opening sequence out of three test sessions

*Received two additional trials

## Discussion

We show that kea were able to solve a rather difficult two-step sequence task and that receiving a demonstration of a skilled conspecific had a positive effect on solving success. However, successful birds showed a high variation in their response topography and often abandoned faithfully copying the task in favour of exploration. This is particularly interesting in the case of John, who followed the demonstrated sequence twice and then generalised in the other direction.

The effect of demonstration becomes particularly clear when combining the results from phase two and three. Eight out of 10 subjects that came to solve the task did so after a demonstration. That kea can profit from social learning is in line with previous studies (Huber et al. 2001; Huber 2002; Suwandschieff et al. 2023). However, the current study provides little evidence for motor imitation, despite various methodological differences from the other studies. For instance, previous experiments illustrated that the motivation to follow a demonstration was low. It was theorized that the complexity of the demonstrated actions could have contributed, as they likely were too simple to require a demonstration to solve the task (Suwandschieff et al. 2023), or too complex to follow the demonstration from afar (Huber et al. 2001) and reproduce with high fidelity. Therefore, the task was made more difficult than the basic two-choice task, while avoiding the complexity of the multi-lock box, and the forced failure in phase one was introduced to prime individuals on the task difficulty and to increase their motivation to follow a demonstration. Yet, these measures did not result in a higher copying fidelity than the other studies. On the one hand, half of the solvers used the opposite sequence to the demonstrators. On the other hand, solving success appeared not to reference solving consistency, as successful birds continued to explore the solving potential by applying different opening methods (see supplementary material for details). This variation in solving behaviour unfortunately made a statistical comparison not possible in terms of actual mechanisms, or quantifiable behavioural differences with previous studies.

As all individuals, regardless of the experimental group, participated in the forced failure task, it is improbable that our results can be solely explained by social facilitation or local enhancement. Although we cannot rule out the possibility that the mere presence of a demonstrator motivated individuals to engage in the task, we have established that all individuals will do so even in the absence of a conspecific. Therefore, the presence of another individual does not appear to be the primary factor explaining our results. Additionally, since the location of the test box remained constant throughout the different phases, no additional information could have been gained from the demonstration. Consequently, this does not explain the discrepancy between the experimental groups. While the success rate of observer birds and the varied response patterns exhibited by successful individuals indicate emulation, we cannot dismiss the potential influence of stimulus enhancement. A test setup that clearly distinguishes these two mechanisms would have to be devised, i.e., one including a ghost control (Whiten and Ham 1992). Consistent with previous findings kea show high behavioural flexibility (Werdenich and Huber 2006; Auersperg et al. 2011; Laschober et al. 2021) and preferentially engage in exploratory behaviour, being more interested in potential affordances than feeding success (Diamond and Bond 1999; Huber et al. 2001; Smith et al. 2022; Suwandschieff et al. 2023). These results are in accordance with kea’s natural feeding strategies, as opportunistic group foragers, with kea paying close attention to what others feed on while engaging in individual manipulation strategies to obtain the resources (Diamond and Bond 1999). In addition, it corresponds with the characteristics of island-dwelling parrots, as described by Mettke-Hofmann and colleagues (2002) who found that island species spend significantly more time on exploratory behaviour especially in areas of seasonally fluctuating food availability.

In conclusion, we find strong evidence that observing a conspecific opening an apparatus via two steps affected the solving success of observer kea. They thus profit from social learning, which aligns well with other studies on parrots and songbirds showing social information transmission and the spread of novel foraging techniques within captive groups and wild populations (e.g. Slagsvold and Wiebe 2011; Auersperg et al. 2014; Aplin et al. 2015; Klump et al. 2021). However, in our study, the response topography of solvers was variable and the copying fidelity was at a very low level, providing no indication of motor imitation over emulation or stimulus enhancement in kea. Therefore, our findings corroborate that kea display strong behavioural variability when attempting to solve a complex motor task. They also keep on exploring options after a successful solution and may rapidly shift solving strategies. Taken together, this makes kea a great model system to study behavioural flexibility but not so much for imitation.

### Supplementary Information

Below is the link to the electronic supplementary material.

Supplementary file1 (PDF 574 KB)

Supplementary file2 (XLSX 60 KB)

Supplementary file3 (MOV 212979 KB)

Supplementary file4 (MOV 115808 KB)

## Data Availability

All data generated or analysed during this study are included in this published article [supplementary material file: raw data].
