# Supplementary material for: Kea, bird of versatility. Kea parrots (Nestor notabilis) show high behavioural flexibility in solving a demonstrated sequence task
Source: J Ornithol. 2023 Dec 16;165(1):49–55. doi: 10.1007/s10336-023-02127-y (PMC10787887; doi:10.1007/s10336-023-02127-y)
Supplement: Supplementary file 1 — Supplementary file1 (PDF 574 KB) [file 10336_2023_2127_MOESM1_ESM.pdf]

Supplementary material: Kea, bird of versatility. Kea parrots (*Nestor notabilis*) show high behavioural flexibility in solving a demonstrated sequence task.

Elisabeth Suwandschieff, Ludwig Huber, Thomas Bugnyar, Raoul Schwing

Methods

Subjects

The kea flock at the research station Haidlhof comprised of 23 individuals with 20 individuals available for testing. Breeding season plays a big role in availability to participate and motivation levels. Consequently, two high ranking non-breeding males were assigned as demonstrators. One female (Wy) had to be excluded from testing due to extended breeding and nesting behaviour. A second female (Ma) had to be excluded from testing as she was unwilling to participate. In total 18 individuals (eight females and ten males) were tested. All individuals were separated into four experimental groups: two control groups, and two test groups, for details see *Table 1*

Table 1 Test subject identification (ID), sex, age, rearing method (hand vs. parent), experimental group assigned, group number and demonstrator assigned (only applicable for Test Groups). Individuals marked with an asterisk in front of their ID were involved in breeding and had to be tested at a later point. Individuals marked with two asterisks after their ID had visual access to the testing compartment. Individuals marked in red dropped out of testing.

#	ID	Sex	Sex (m/f)	Age	Raised	Experimental Group	Group Number	Demonstrator
1	Fr	♂	m	17	parent	Control	1	Dem
2	Pa	♂	m	11	parent	Control	1	Dem
3	Di	♀	f	4	hand	Control	1	NA
4	Sy	♀	f	14	hand	Control	1	NA
5	Sk	♂	m	4	hand	Control	1	NA
6	Ke	♂	m	17	hand	Control	2	NA
7	*Fy**	♀	f	5	parent	Control	2	NA
8	Pl	♀	f	14	hand	Control	2	NA
9	*Od**	♂	m	6	hand	Control	2	NA
10	Pu	♀	f	8	hand	Control	2	NA
11	Ti	♀	f	3	parent	Test	3	Fr
12	*Je**	♂	m	6	hand	Test	3	Fr
13	Jo	♂	m	22	parent	Test	3	Fr
14	Ma	♀	f	8	parent	Test	3	Fr
15	Wy**	♀	f	14	hand	Test	3	Fr
16	Co	♀	f	14	hand	Test	4	Pa
17	Pi	♂	m	17	hand	Test	4	Pa
18	Ro	♂	m	13	parent	Test	4	Pa
19	Pn	♂	m	4	hand	Test	4	Pa
20	Ly**	♀	f	14	hand	Test	4	Pa

Schematic set-up

All tests were performed in the testing compartment *Porticuli Aurorae* of the aviary, with *Porticula Exspectationis* serving as the observation and *Porticula Rerum* as the demonstration and test compartment, see *Figure 1*. The demonstration was performed with the test box facing the observer birds at the same angle and side they approached during their test. This set-up allowed the observers to remain put while the demonstrators entered and exited the demonstration/test compartment during the session. Demonstrators were not present during observer bird testing.

Two four-meter planks were placed directly in front of the test box at a distance of one meter in the observation compartment, marking the observation area for the video camera. One camera was setup outside the aviary directly opposite the test box overlooking the marked observation area (planks). A second camera (GoPro) was mounted to the test box directly (on a stick) in order to get a birds-eye-view of the test. Both demonstration and test sessions were recorded.

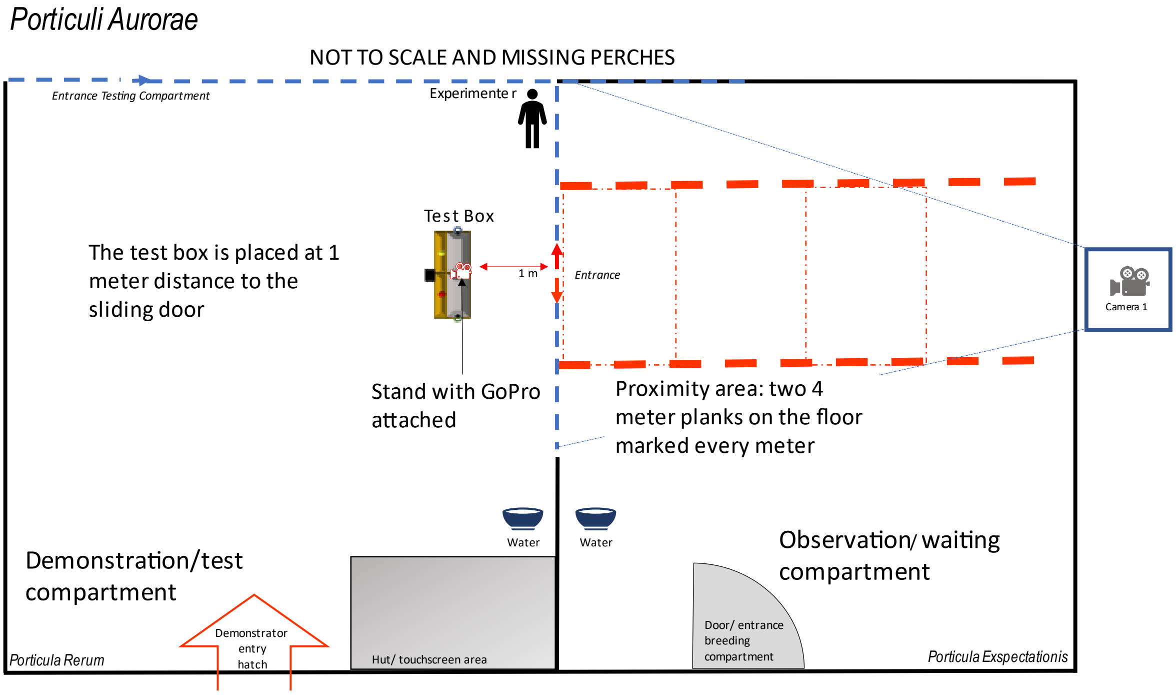

Figure 1 Schematic of kea testing compartment set up: split into demonstration/test and observation/waiting compartment, marked proximity area, camera position outside the aviary, camera position on top of the test box, entrance demonstrators (big red arrow) and experimenter position.

Habituation and training procedure

All birds received test box habituation in the demonstration/test compartment. They were allowed to freely feed from a dummy box (visually identical to the test box) with all movable parts (pins, rings and sliding lid doors) and electronics missing. Both sides of the test box

were baited and criterion to complete habituation was successful feeding in minimum of 3 consecutive sessions (1 session à 1 feeding bout). Habituation was also used to correct for any potential side biases. For this purpose, the dummy box was placed at different positions in the test compartment to make sure both sides were approached and fed from.

Demonstrators received additional demonstration training on their side/ sequence. Each demonstrator was assigned one pin and one ring and was trained on one side only with either sequence red pin – blue ring or yellow pin – gree ring. Demonstrator training comprised of four phases. In the first phase, only the assigned pin and ring were present and the opposite side was locked. In the second phase, two pins (one assigned and one not assigned) and assigned ring were present (the opposite side was locked). In the third phase, all pins and rings were present and the box was not locked (all responses technically possible). In the final phase, the test box was moved around the compartment to ensure reliable demonstration even when the test box is approached from different angles and sides. The minimum requirement to complete each phase was successfully solving the task (without hesitation or touching any other parts) in three consecutive sessions with three trials each. Once demonstrator training was completed the first Test Group was tested.

Hypothesis and predictions

Phase 2: Non-demonstrated task Control Groups (CG)

The following hypothesis and predictions were made about condition two:

H1: Kea can learn to solve a sequence task

P1.1: Control Group individuals will learn to solve the sequence task via trial and error.

P1.2: Once an individual has learned to solve the task they will develop a bias in favour of one side/sequence (win-stay strategy).

Phase 2: Demonstrated task Test Groups (TG)

The following hypothesis and predictions were made about condition two:

H2: Kea will imitate a demonstrator solving a sequence task.

P2.1: Test Group individuals will preferentially use the demonstrated opening side sequence and colour to solve the task.

P2.2: Test Group individuals will be better at solving the task than the Control Group individuals, as visible in the number of successful sessions.

P2.3: Once an individual has learned to solve the task they will develop a bias in favour of one side/sequence (win-stay strategy) even if this is not the demonstrated side.

Phase 3: Demonstrated task Control Test (CT)

The following hypothesis and predictions were made about condition three:

H3: Kea will learn to solve the task by watching a trained demonstrator.

P3.1: Kea that had previously not received a demonstration will learn to solve the task by watching a demonstration.

P3.2: Control Test Group individuals will be faster at solving the task, as visible in the first session, with a reduced approach duration and response latency as compared to their previous results as Control Group individuals.

P3.3: More individuals will be able to solve the task after watching a trained demonstrator than through trial and error.

Data scoring

All experiments were videotaped from two sides, behind the observation compartment and directly above the test box within the test compartment (GoPro). All GoPro videos of the test sessions were scored/coded (excluding the observation only sessions) with Solomon Coder (version beta 19.08.02). In two cases the video footage from behind the observation compartment had to be coded as the SD card in the GoPro failed and the session was not recorded. The approach duration (from opening of gate to touching any part of to test box), response duration (from first touch of the test box to feeding response or time out) the solving latency (from the first touch of any part of the test box to the feeding response after successfully solving the task) and, response type frequency (touching red or yellow pin, pulling red or yellow pin, touching blue or green ring, pulling blue or green ring, feeding) was coded for all test sessions. One independent rater, who was blind to the study, scored 10% of all videos (assigned at random) to check for the interobserver reliability, with Kapa for the categorical and Intraclass Correlation Coefficient for the numerical data.

Cohen's Kappa was at near perfect agreement (k = 0.95) for all response type frequencies: touching red or yellow pin, pulling red or yellow pin, touching blue or green ring, pulling blue or green ring, feeding. The Interclass Correlation Coefficient for durations and latencies was at moderate agreement for approach duration and at excellent agreement for response duration and solving latency (ICC = 0.711, approach duration; ICC = 0.999, response duration; ICC = 1, solving latency).

Additional materials

Kea photos

(a) (b)

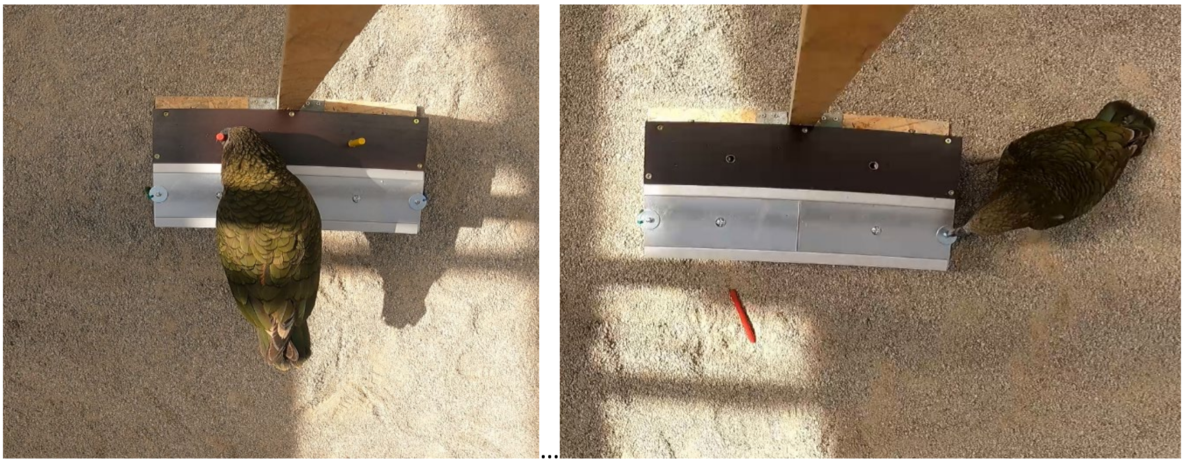

Figure 2 kea attempting to solve the task (GoPro view), (a) pulling the red pin (b) moving towards pulling the blue ring. Both pins have been pulled in this example and therefore the test box is locked.
